# Ayurvedic Therapeutic Regimen as an Add-On to Optimized Conventional Management of Parkinson Disease: Protocol for an Exploratory Randomized Controlled Trial Evaluating Clinical, Cortical Excitability, Neuroimmune, and Autonomic Function Parameters

**DOI:** 10.2196/83336

**Published:** 2026-01-09

**Authors:** Umesh Chikkanna, Pramod Kumar Pal, Sophia Jameela, Rohan R Mahale, Nitish Kamble, Venkataram Shivakumar, Bharath Holla, Ravi Yadav, Vikram V Holla, Talakad Narasappa Sathyaprabha, Kaviraja Udupa, Srinibash Sahoo, Manjunath M Venkataswamy, Selva Ganapathy, Chandra Shekara Rao, Babita Yadav, Sreenithya M, Shivarama Varambally, Sulochana Bhat, Narayanam Srikanth, Kishore Kumar Ramakrishna

**Affiliations:** 1 Department of Integrative Medicine, National Institute of Mental Health and Neurosciences, Hosur Main Road, Bengaluru, 560029, India, 91 797585661; 2 Department of Neurology, National Institute of Mental Health and Neurosciences, Bengaluru, India; 3 Central Council for Research in Ayurvedic Sciences, New Delhi, India; 4 Department of Neurophysiology, National Institute of Mental Health and Neurosciences, Bengaluru, India; 5 Central Ayurveda Research Institute, Bengaluru, India; 6 Department of Neurovirology, National Institute of Mental Health and Neurosciences, Bengaluru, India; 7 Department of Physiotherapy, National Institute of Mental Health and Neurosciences, Bengaluru, India; 8 Department of Psychiatry, National Institute of Mental Health and Neurosciences, Bengaluru, India

**Keywords:** Ayurveda, Parkinson disease, transcranial magnetic stimulation, autonomic function test, immunoparameters

## Abstract

**Background:**

Parkinson disease (PD), a progressive neurodegenerative disorder, lacks disease-modifying treatments. Current therapies focus on symptomatic relief, highlighting the need for adjunctive neuroprotective strategies. Ayurveda, a holistic system, shows promise in improving PD clinical outcomes.

**Objective:**

This assessor-blinded randomized controlled study aims to systematically evaluate the efficacy of an add-on Ayurveda therapeutic regimen compared to conventional treatment as usual (TAU) in improving the clinical outcomes of PD.

**Methods:**

A total of 80 patients with PD, diagnosed according to UK Parkinson’s Disease Society Brain Bank criteria, will be randomized into two groups: TAU and add-on Ayurveda. The intervention group will receive Ayurvedic therapy alongside conventional treatment for 180 days, whereas the control group will continue TAU. Assessments will occur at baseline and at 60-, 120-, and 180-day follow-ups, evaluating motor and nonmotor symptoms. Transcranial magnetic stimulation, heart rate variability, and pulmonary function tests will assess cortical excitability, autonomic function, and pulmonary function, respectively. Immunological parameters, including cytokine levels and telomere length, will be analyzed at baseline and at 180 days to explore disease-modifying effects. Liver and renal function tests will monitor safety.

**Results:**

As of 2025, a total of 259 patients with PD have been screened for eligibility, of whom 58 participants have been successfully enrolled in the trial. Among these, 33 participants have completed the intervention and follow-up assessments, 14 have discontinued participation, and 11 are currently continuing participation in the study. Recruitment and follow-up are ongoing, and the trial is scheduled for completion in September 2026.

**Conclusions:**

This study aims to address the lack of mechanistic evidence and robust data on Ayurveda in PD. By systematically evaluating clinical efficacy and potential biomechanisms, the findings will provide preliminary evidence for Ayurvedic interventions, potentially paving the way for their integration into comprehensive PD management.

## Introduction

### Background

Parkinson disease (PD), a progressive neurodegenerative disorder characterized by motor symptoms such as tremor and rigidity, affects diverse populations worldwide [1]. First described in 1817, PD’s prevalence increases with age, impacting approximately 1% of individuals aged >65 years [2,3]. Regional variations in prevalence exist. Indian studies show a crude prevalence of 14.1 per 100,000 in rural Kashmir, with rates rising to 247 per 100,000 in those aged >60 years [4]. Conversely, Bangalore reported a lower crude prevalence of 27 per 100,000 [3]. Notably, a higher rate of 328.3 per 100,000 was observed among the Parsi community in Mumbai [5]. Early-onset PD, occurring before the age of 40 years, accounts for 3% to 5% of cases, and PD is generally twice as common in men [6]. Beyond motor symptoms, PD presents nonmotor challenges such as sleep disturbances and cognitive impairment. Autonomic dysfunction also increases with age and disease progression. Patients face a 6-fold increased risk of dementia [7].

Standard PD treatment focuses on symptom management using medications such as levodopa and dopamine agonists [8]. However, these treatments do not halt disease progression and can cause side effects [9,10]. Invasive procedures such as deep-brain stimulation are expensive and inaccessible to many, particularly in India. Consequently, many patients with PD explore complementary and alternative medicine (CAM), including Ayurveda [11]. Studies indicate that nearly half of patients with PD use CAM, typically educated, younger, urban individuals with longer disease duration [11].

While CAM, including Ayurvedic interventions, shows potential for improving motor function, robust clinical evidence is lacking. Existing research consists of limited clinical trials, pilot studies, and case reports. A systematic review of published clinical trials evaluating Ayurvedic interventions in PD suggests that therapies such as Mucuna pruriens and various Panchakarma procedures may offer potential benefits in symptom management [12]. However, the evidence is insufficient to reach any reliable conclusion. There is a need for randomized controlled trials (RCTs) with objective measures to evaluate the mechanisms of Ayurvedic treatments. Specifically, the impact of comprehensive Ayurvedic therapies on cortical activity, neuroimmune function, autonomic function, telomere length, and neuropsychological function remains unexplored. Investigating these areas will provide crucial evidence for the efficacy and safety of Ayurveda in PD and shed light on the neurobiological basis of its effects.

### Ayurveda and PD

PD can be comprehended within the paradigm of Vāta Vyādhi (neurological disorders) as delineated in Ayurveda. It is primarily attributed to Vāta Dosha Prakopa (vitiation of Vata Dosha, a bodily humor that is primarily associated with movement), which manifests as tremors, rigidity, and bradykinesia. The chronicity and progressive nature of the disease necessitate a multidimensional therapeutic approach involving Vāta-pacifying strategies, Rasāyana (rejuvenating measures), and Panchakarma (detoxification therapies) for symptomatic relief and improving disease progression [13].

### Objectives

This assessor-blinded randomized controlled study aims to systematically evaluate the efficacy of an add-on Ayurveda therapeutic regimen compared to conventional treatment as usual (TAU) in improving the clinical outcomes of PD.

The secondary objectives are to explore the effects of add-on Ayurveda therapy in comparison to conventional TAU on multiple physiological and functional parameters, namely cortical excitability, assessed through single (motor threshold [MT], resting cortical, and silent period [SP]) and paired pulse (short intracortical inhibition [SICI], long-interval cortical inhibition [LICI], and intracortical facilitation [ICF]) transcranial magnetic stimulation (TMS) measures; and immune parameters, including Th1/Th2/Th17/T-regulatory cell population, plasma C-reactive protein, plasma levels of Th1/Th2/Th17 pathway cytokines, and telomere length.

### Hypothesis

This study hypothesizes that Ayurveda treatment, when integrated with standard TAU, will be safe and effective in improving clinical and cognitive outcomes in patients with idiopathic PD. It is expected that these therapies will enhance the optimal response to levodopa, potentially leading to better symptomatic control. Ayurveda interventions are hypothesized to modulate cortical excitability measures, which will correlate with improvements in both clinical and cognitive parameters. Furthermore, it is postulated that add-on Ayurveda therapies may induce a significant shift in the Th1/Th2/Th17/T regulatory cell population, along with alterations in plasma levels of pro-inflammatory cytokines and telomere length, which will further correlate with clinical, cognitive, and neurophysiological outcomes. Finally, it is anticipated that autonomic dysfunction, as measured through heart rate variability (HRV), will show significant improvement following Ayurveda interventions, aligning with overall clinical benefits observed in patients with PD.

## Methods

### Study Design Description

This study is designed as an assessor-blinded RCT to be conducted at the National Institute of Mental Health and Neurosciences (NIMHANS), Bengaluru. The study protocol was developed collaboratively by investigators from the NIMHANS and the Central Council for Research in Ayurvedic Sciences (CCRAS) based on existing clinical and preclinical evidence, and it underwent expert peer review before funding approval.

### Trial Design

**Figure 1.**
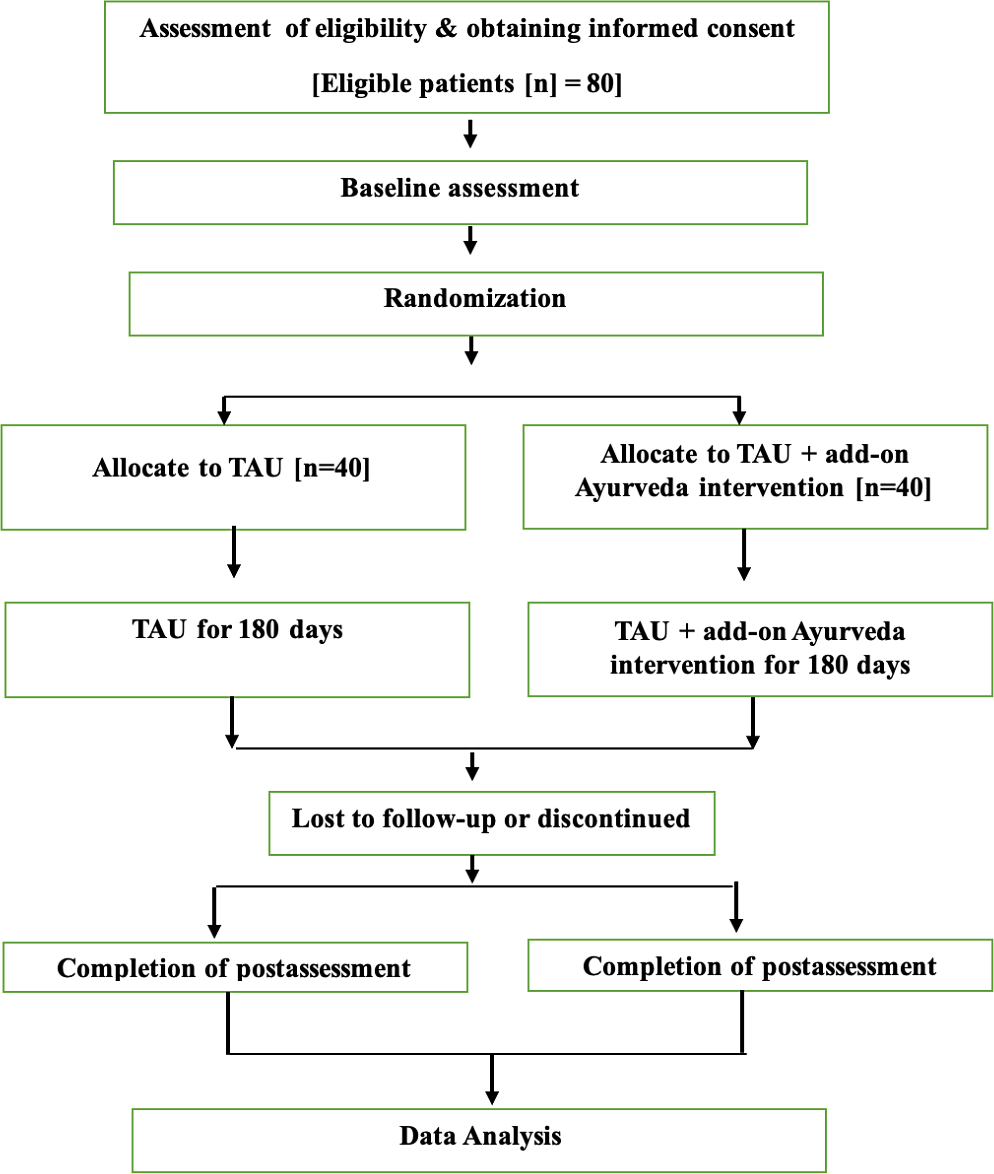
Study design. TAU: treatment as usual.

This study is a prospective, assessor-blinded, exploratory RCT with a parallel-group design and a 1:1 allocation ratio. It is designed as a superiority trial to evaluate the potential benefits of an Ayurvedic therapeutic regimen as an add-on to optimized conventional management in patients with PD, compared to optimized conventional management alone. The trial will be conducted at a single tertiary care center (NIMHANS, Bengaluru, India) over a period of 3 years (Figure 1).

Participants will be followed for 24 weeks post-randomization. The study aims to generate preliminary evidence on clinical efficacy and mechanistic insights related to cortical excitability, neuroimmune function, and autonomic function. Participants will be randomized into either the add-on Ayurveda intervention arm or the TAU control arm, with each participant undergoing a 6-month study period.

Participants will be recruited from both the outpatient department (OPD) and inpatient department services of the Departments of Neurology and Integrative Medicine at the NIMHANS, Bengaluru, India. NIMHANS is a tertiary care neuropsychiatric and neurosciences hospital and Institute of National Importance under the Ministry of Health and Family Welfare, Government of India. The study will be conducted within NIMHANS using its specialized neurology and integrative medicine infrastructure for recruitment, intervention delivery, and follow-up assessments.

Those meeting the inclusion criteria will be recruited after obtaining written informed consent. Informed consent will be obtained by the senior research fellow (SRF) possessing an MD (Ayurveda) qualification, who has been trained in Good Clinical Practice (GCP) [14] and institutional ethical guidelines for human research. The study procedures, potential benefits, risks, and the right to withdraw at any stage will be clearly explained in the patient’s language, and a copy of the consent document will be provided (Multimedia Appendix 1).

To promote adequate enrollment, participants will be recruited through advertisements in print and institutional media, as well as by prescreening of hospital health records to identify potentially eligible individuals. Efforts will also be made to minimize participant burden by providing clear information, convenient scheduling of assessments, and coordinated support from study staff during recruitment and follow-up.

### Selection of Participants

#### Inclusion Criteria

Participants eligible for the study will be those diagnosed with Idiopathic PD based on the UK Parkinson’s Disease Society Brain Bank criteria [15]. The study will include individuals in Hoehn and Yahr stages 2.5 to 4, with a disease duration of more than 3 years, experiencing severe motor fluctuations and suboptimal response to standard treatment, including dyskinesia and gait freezing. The eligible age range for participation will be 40 to 70 years. Participants must be on a stable dopaminergic regimen for ≥2 weeks before baseline.

#### Exclusion Criteria

Participants with Parkinsonism spectrum disorders or other neurodegenerative conditions, such as motor neuron diseases and multiple sclerosis, will be excluded from the study. Individuals with major cardiovascular disorders or those with hepatic, renal, or uncontrolled pulmonary dysfunction will not be eligible. Patients receiving concomitant medications that may interfere with Parkinsonian symptoms, including anticoagulants, neuroleptics, metoclopramide, Compazine, beta-blockers, fluoxetine, clozapine, quetiapine, olanzapine, buspirone, antipsychotics, and central nervous system stimulants such as sodium valproate, will also be excluded. Those with cognitive impairment, defined by a Mini-Mental State Examination score of <18, as well as individuals with evidence of malignancy or a history of substance abuse, will not be considered for participation. Additionally, patients who have participated in any other clinical trial within the past 6 months will be excluded to ensure unbiased study outcomes.

### Sample Size

This study has been designed as an exploratory trial primarily aimed at assessing feasibility and generating preliminary clinical evidence on the effects of a multicomponent Ayurvedic add-on therapy in PD. The sample size estimation was based on parameters from a prior exploratory study [16], which reported a mean change in the Unified Parkinson’s Disease Rating Scale (UPDRS) score of 1.0 with an SD of 2.0 and a correlation coefficient of 0.60. Using these assumptions, a 2-tailed alpha of 0.05, and 80% power, the required sample size was calculated to be 28 participants per group. Allowing for a 20% attrition rate, a total of 40 participants per group (80 overall) will be recruited.

This sample size is expected to provide preliminary insights into the effects of add-on Ayurveda therapy in PD and to serve as a basis for future large-scale confirmatory trials.

### Assignment of Interventions

Participants will be prescreened from the OPD and inpatient department of consultant neurologists at NIMHANS by the designated study team. Following prescreening, the principal investigator (PI) will conduct formal screening procedures and enroll eligible participants. Allocation to study arms will be performed using a computer-generated randomization sequence prepared by an independent statistician from the funding agency (CCRAS). Allocation concealment will be ensured using the sequentially numbered, opaque, sealed envelopes approach to prevent selection bias during participant enrollment. Once the patient is enrolled, the study PI shall provide the sequentially numbered envelope with the group allocation concealed inside to the participant who will open it.

The Ayurvedic interventions will be administered by the PI, who is a qualified Ayurveda physician. Standard conventional care will continue to be managed by the participant’s treating neurologist, as per routine clinical practice. However, the assessor conducting the assessments will remain blinded to group allocation to minimize bias.

### Intervention

The interventions used in this study consist of two arms: group A (control group) receiving conventional TAU and group B (intervention group) receiving add-on Ayurveda therapy along with TAU of PD, prescribed by the treating neurologist.

#### Group A (Conventional TAU—Control Group)

Participants will receive pharmacological treatment as prescribed by the treating neurologist. This would include tablet levodopa and carbidopa 125 mg or tablet entacapone 200 mg or tablet pramipexol 1 mg or tablet selegiline 5 mg as appropriate per the assessment of the consultant neurologist.

#### Group B (Conventional TAU With Add-On Ayurveda—Intervention Group)

Participants will receive Ayurveda intervention as an adjunct to the conventional treatment for a period of 180 days, comprising 3 cycles of Ayurveda therapies at 60-day intervals. The regimen is structured as follows:

- Preparatory phase (*Deepana-Pachana* therapy): To enhance digestion and metabolism, participants will be administered *Chitrakadi Vati* (250 mg per tablet), 2 tablets with warm water, 3 times daily before food for a duration of 5 days.
- Shodhana therapy (purification phase—*Basti* [rectal administration of medicine] therapy): This includes a Yoga Basti regimen, wherein a combination of *Niruha Basti* (therapeutic decoction enema) and *Anuvasana Basti* (oil enema) will be administered in a prespecified regimen in the form of *Basti* comprising one *Sneha Basti* initially, 3 decoction *Bastis* alternately followed by 3 *Sneha Bastis,* and 1 *Sneha Basti* in the end aimed at pacifying *Vāta Dosha*.
  - Niruha Basti: Decoction of *Erandamoola* (made with the root of *Ricinus Communis* Linn [450 mL]) administered rectally on an empty stomach as per the protocol for *Yoga Basti*.
  - *Anuvasana Basti*: *Bala Taila* (90 mL) administered immediately after food on 5 occasions as per the protocol for *Yoga Basti*.
- Shamana therapy (pacificatory phase): Following purification, the participants will receive oral Ayurveda formulations for symptomatic relief and alleviation of vitiated *Vata Dosha* for 47 days in each cycle as follows:
  - *Mashabaladi Kwatha* (decoction)—15 mL, with 2 pinches of asafoetida and rock salt administered with warm water 3 times daily.
  - *Kalyanaka Ghrita* (medicated ghee)—20 mL, taken early in the morning on an empty stomach.
- The complete Ayurveda intervention regimen will be repeated thrice over the study period, with 3 cycles of 60 days each, totaling 180 days.

Usual concomitant care (if any), including physiotherapy and occupational therapy, will be permitted as part of standard management and will be recorded as concomitant therapy. However, the use of any additional Ayurvedic therapies or herbs not specified in the study protocol will be prohibited during the trial to avoid confounding effects. All concomitant medications, including any dose modifications of standard medications or rescue interventions required for symptom management, will be documented at each study visit in the case report forms.

### Strategies for Improving the Fidelity of Care Providers and Adherence of Participants

The Ayurvedic intervention is standardized and will be administered uniformly as per the protocol. Participants will be screened for suitability before Panchakarma therapy, and the intervention will be withheld in cases of intolerance, fever, respiratory infection, or any other clinical contraindication. To ensure uniformity, all procedures will follow standard operating protocols, and participants will receive patient information leaflets detailing the interventions.

As panchakarma therapies are delivered in an inpatient or closely supervised setting by trained professionals, adherence will be monitored daily through direct supervision and treatment logs. Participants are required to maintain a compliance form to record the intake of both standard care and add-on oral Ayurvedic medications, which will be reviewed by the study team during follow-up visits. Furthermore, to promote participant retention and minimize missing data, follow-up will be reinforced through periodic telephonic contact between scheduled visits.

### Outcome Measures

The primary outcome is the change in the total score of the Movement Disorder Society-UPDRS (MDS-UPDRS) after 6 months of treatment, compared with the baseline. MDS-UPDRS Part III will be assessed in both the ON and OFF medication states. The OFF state assessment will be conducted after a 12-hour withdrawal from the patient’s last dopaminergic medication, whereas the ON state assessment will be performed 1 hour after the administration of the first morning dose of dopaminergic medication. The OFF-state evaluation (after 12 h withdrawal) reflects the underlying disease severity, whereas the ON-state evaluation (1-h postmorning dose) reflects treatment responsiveness. The total MDS-UPDRS change is thus intended to integrate both clinical states, which is relevant for this exploratory phase of the Ayurvedic add-on intervention assessment.

The secondary outcome measures include a range of neurophysiological, biochemical, cognitive, and functional parameters. Cortical excitability will be assessed using TMS, evaluating both single-pulse (MT, resting cortical, and SP) and paired-pulse (SICI, LICI, and ICF) measures. Autonomic function will be analyzed through HRV, whereas pulmonary function tests will assess respiratory capacity.

Exploratory mechanistic outcomes include alterations in immunological markers, including changes in Th1/Th2/Th17/T regulatory cell populations, plasma levels of pro-inflammatory cytokines, and telomere length, which will be measured to evaluate the immunomodulatory and anti-inflammatory effects of the intervention.

Functional and motor outcomes will be examined through reductions in dyskinesia severity using the Unified Dyskinesia Rating Scale and improvements in gait parameters, measured using the 6-minute walk test and gait speed (10-m walk test). Cognitive function will be assessed using the Montreal Cognitive Assessment, and postural stability will be evaluated through the Balance Error Scoring System. Changes in Scales for Outcomes in Parkinson’s disease-Sleep will be monitored to assess improvements in sleep disturbances associated with PD.

Safety assessments will include monitoring for treatment-emergent adverse events throughout the study period. Liver function tests, renal function tests, and complete blood count will be performed at baseline and at the end of the study (day 180) to detect any potential systemic adverse effects. For participants undergoing Basti therapy, adverse events (AEs) will be monitored during the in-patient care.

Participants in the TAU arm will continue with their dopaminergic medications. At baseline, stabilized or optimized dopaminergic doses will be recorded, and any subsequent changes during the trial will be documented along with dates and reasons. Levodopa equivalent daily dose (LEDD) will be calculated at each visit (baseline and follow-ups) using standardized conversion factors and tracked longitudinally.

All clinical and neurophysiological outcome assessments will be performed by a trained SRF with an MD (Ayurveda) qualification, and the routine assessments will be done by the neurologists involved in the study before prescribing the study TAU interventions or deciding changes in interventions. The SRF underwent a 3-month structured training program under the supervision of a neurologist and neurophysiologist to ensure competency in administering both clinical and neurophysiological assessments. The same SRF will also be responsible for data collection throughout the study to maintain consistency.

All outcome measures are evaluated using validated clinical scales and standardized laboratory instruments, as detailed in the manuscript, each with established reliability and validity. Before the commencement of data collection, all laboratory instruments and procedures will be calibrated and standardized. To promote data quality, assessors received formal training, and interrater reliability for the primary outcome measure (MDS-UPDRS Part III) will be established in at least 20% of participants.

### Ethical Considerations

#### Overview

The trial has been approved by the NIMHANS Human Ethics Committee for Research in AYUSH and Integrative Medicine (NIMHANS/HECAIM/5th/MEETING/2021‐2022, dated January 7, 2022). All procedures in the trial will comply with the ethical guidelines outlined by the Indian Council of Medical Research and the Declaration of Helsinki [14,17]. Participants will be recruited only after obtaining written informed consent, ensuring that they fully understand the study’s objectives, interventions, potential risks, and benefits. The study is covered by trial insurance from the Oriental Insurance Company. Confidentiality of the study participants will be ensured by anonymization of the data.

#### Consent

Informed consent will be obtained by the SRF possessing an MD (Ayurveda) qualification, who has been trained in GCP [14] and institutional ethical guidelines for human research. The SRF has prior experience in conducting clinical studies and in obtaining informed consent in accordance with ethical standards.

The consent process will be conducted in the participant’s preferred language, ensuring adequate explanation of study objectives, procedures, potential risks, and benefits. Ample time will be provided for participants to ask questions and decide about participation voluntarily. The entire process will be documented, and a copy of the signed consent form will be provided to each participant.

Any AEs occurring during the course of the trial will be promptly reported to the Data and Safety Monitoring Board (DSMB) as per the approved protocol. Participants experiencing an AE will receive appropriate and immediate clinical care. Each event will be independently reviewed by a committee comprising experts from both the Ayurveda system of medicine and modern medical sciences.

If, upon thorough evaluation, the AE is determined to be related to the investigational intervention, compensation will be provided to the participant in accordance with regulatory requirements through the study’s approved insurance coverage.

### Brief Description of Some Relevant Assessments

#### Clinical Assessments

##### Movement Disorder Society–Unified Parkinson’s Disease Rating Scale

This scale assesses motor and nonmotor PD symptoms. It includes four parts: nonmotor experiences of daily living (part I), motor experiences of daily living (part II), motor examination (part III), and motor complications (part IV). Parts IA, IB, and II are conducted in the “ON” state, whereas Part III is assessed in both “ON” and “OFF” states. Part IV integrates patient-reported information with clinical observations [18].

##### Unified Dyskinesia Rating Scale

This tool evaluates involuntary movements (dyskinesia) associated with PD treatment. It includes historical (on-dyskinesia and off-dystonia) and objective (impairment and disability) sections, focusing on choreic and dystonic movements [19].

##### Scales for Outcomes in Parkinson’s Disease–Sleep

This assesses nighttime and daytime sleep problems over the past month. It includes subscales for nighttime sleep and daytime sleep, with a global assessment of nocturnal sleep quality [20].

##### Montreal Cognitive Assessment

This tool evaluates cognitive function, assessing memory, language, executive functions, visuospatial skills, attention, abstraction, and orientation. It provides a Derived Memory Index score to predict Alzheimer dementia conversion [21,22].

##### 10-m Walk Test and 6-Minute Walk Test

These assess gait. The 10-m walk test measures gait speed, whereas the 6-minute walk test evaluates aerobic capacity and endurance. Both are performed in the “OFF” state [23,24].

##### Balance Error Scoring System

This measures postural stability using the Biodex balance system. It involves 3 stances (double-leg, single-leg, tandem) performed on firm and foam surfaces with eyes closed. Errors are scored, with lower scores indicating better balance [25].

##### Transcranial Magnetic Stimulation

This noninvasive technique assesses motor cortical function. TMS is conducted through “Magventure R30 with magoption” equipment using the MCB-70 coil. It measures parameters such as resting MT (RMT), central motor conduction time, SP, SICI, LICI, and ICF. It is performed in the “OFF” state [26].

##### Heart Rate Variability

This assesses autonomic function using the “Chronovisor” HRV machine. Time-domain (SDNN, pNN50, RMSSD) and frequency-domain (low frequency, very low frequency, ultra low, high frequency, and low frequency–to–high frequency ratio) measures are acquired in the “OFF” state.

##### Pulmonary Function Test

This evaluates respiratory capacity using spirometry. Lung volume measures (forced vital capacity, forced expiratory volume in 1 s, and forced expiratory volume in 1 s–to–forced vital capacity ratio) and respiratory muscle strength (MIP and MEP) are assessed in the “OFF” state.

### Neuroimmune Parameters

#### Objectives

The objectives are to assess (1) the proportions and activation status of human peripheral blood Th1/Th2/Th17 cells and (b) the estimation of cytokine by Cytometric Bead Array.

#### Peripheral Blood Mononuclear Cell Isolation and Cryopreservation

Peripheral blood mononuclear cells (PBMCs) and plasma samples will be separated from peripheral whole blood samples (10 mL) by density gradient centrifugation and cryopreserved.

### Procedure

#### Staining With Fluorescent Antibodies for Immunophenotyping of Th1/Th2/Th17 Cells

Cryo-preserved PBMCs will be processed for multiparametric assessment of the frequencies of major adaptive immune T cells, namely Th1, Th2, Th17, and Treg cells. Briefly, 1 million PBMCs for each panel of immune surface markers will be stained with LIVE/DEAD Fixable green (Life Technologies) to exclude dead cells and with respective antibodies for 20 and 30 minutes, respectively, at 4°C. Cells will be washed and resuspended in 0.5% paraformaldehyde for fixation. Finally, the cells will be washed and resuspended in phosphate-buffered saline (containing 1% fetal bovine serum). An unstained control will be included with all samples. Stained samples will be acquired using a FACSLyric (BD Biosciences) flow cytometer. Analysis is done by using FlowJo software. Quality control of flow cytometry analysis will be ensured by monitoring the performance of the instrument using Cytometer Setup and Tracking beads. Compensation for fluorescence spectral overlaps will be calculated and applied for the analysis of data for each of the different antibody cocktails. Data will be acquired through the BD FACSuite interface and analyzed on FlowJo software (Table 1).

**Table 1.** Design of fluorescent antibody panel based on the lasers and detection filters available in the FACSLyric flow cytometer for the identification of Th1, Th2, and Th17 cells.

Lasers and antibody marker		Fluorochrome
Violet laser (405 nm)		
Anti-CD4		BV 421
Anti-CD25		BV 605
Anti-CD127		BV 750
Blue laser (488 nm)		
Anti-CCR6[CD 196]		Alexa Fluor 488
Anti-CD8		PE
Anti-CCR4[CD 194]		PE CY 7
Anti-FoxP3		BB 700
Red laser (640 nm)		
Anti-CD3		AF 647
Anti-CXCR3[CD 183]		BD Horizon R 718

#### Estimation of Cytokine by Cytometric Bead Array

Cryopreserved plasma specimens will be processed according to the manufacturer’s instructions for the detection and quantification of the proposed cytokines using the Cytometric Bead Array kit (BD Biosciences). Data will be acquired on FACS Verse flow cytometer (BD Biosciences) and analyzed on FCAP Array software (BD Biosciences Table 2).

**Table 2.** Participant enrollment and schedule of assessment.

Assessments	Baseline	60th day	120th day	180th day
MDS-UPDRS^a^	✓	✓	✓	✓
UDyRS^b^	✓	✓	✓	✓
SCOPA-S^c^	✓	✓	✓	✓
MoCA^d^	✓	✓	✓	✓
6-minute walk test Gait speed (10-m walk test)	✓	✓	✓	✓
BESS^e^	✓	✓	✓	✓
Laboratory parameters				
Hematology				
Complete hemogram	✓	—^f^	—	✓
ESR^g^	✓	—	—	✓
Biochemistry				
Liver function test	✓	✓	—	✓
Renal function test	✓	✓	—	✓
Lipid profile	✓	—	—	✓
HbA_1c_^h^	✓	—	—	✓
FBS^i^	✓	—	—	✓
Sr. Uric acid	✓	—	—	✓
Inflammatory markers				
Plasma cytokine assay	✓	—	—	✓
Immunophenotyping for Th1/Th2/Th17/T-regulatory cell population	✓	—	—	✓
TMS^j^				
Single pulse measures	✓	—	—	✓
Paired pulse measures	✓	—	—	✓
Neurophysiological parameters	✓	—	—	✓
HRV^k^	✓	—	—	✓
PFT^l^	✓	—	—	✓
Concomitant medication	✓	✓	✓	✓
Rescue medication	—	✓	✓	✓

a MDS-UPDRS: Movement Disorder Society–Unified Parkinson’s Disease Rating Scale.

b UDyRS: Unified Dyskinesia Rating Scale.

c SCOPA-S: Scales for Outcomes in Parkinson’s Disease–Sleep.

d MoCA: Montreal Cognitive Assessment.

e BESS: Balance Error Scoring System.

f Not available.

g ESR: erythrocyte sedimentation rate.

h HbA_1c_: glycated hemoglobin.

i FBS: fetal bovine serum.

j TMS: transcranial magnetic stimulation.

k HRV: heart rate variability.

l PFT: pulmonary function test.

### Plans for Data Storage, Handling, and Availability

Research investigators and study personnel will receive comprehensive, documented training on the protocol. Access to CRFs and electronic CRFs will be limited to trained individuals to ensure standardized data entry. MDS-UPDRS scores will be recorded on paper and cross-verified with electronic entries to minimize errors. Participant data will be coded, securely stored with password protection, and assigned unique identification codes for pseudonymization. The data analyst will be blinded to group allocation. PIs will ensure secure data sharing mechanisms for privacy and confidentiality.

Upon completion of the study, the deidentified research data will be submitted to and maintained jointly by CCRAS and the NIMHANS. The dataset will be stored in the research data repository managed by CCRAS. Access to the data can be requested by contacting the funding agency through the official CCRAS website [27] and submitting a formal request describing the intended use. Data will be shared upon reasonable request, subject to institutional approvals and applicable data sharing regulations. No public code repository is applicable for this protocol.

### Statistical Analysis

All analyses will be performed using SPSS software. The primary outcome, change in MDS-UPDRS Part III score from baseline to 24 weeks, will be analyzed using a mixed model for repeated measures with treatment, time, and treatment×time interaction as fixed effects and baseline values as covariates. LEDD will be included as a time-varying covariate to account for dopaminergic medication effects. Results will be reported as least-square mean differences with 95% CIs. The main analysis will follow a modified intention-to-treat approach including all randomized participants who receive at least one dose of the assigned intervention, whereas a per-protocol analysis will be performed as a sensitivity check on participants who complete ≥80% of scheduled therapies and medication adherence.

Missing data will be handled under the assumption of data missing at random using the mixed model for repeated measures model, which uses all available data without imputation. Additional sensitivity analyses, including multiple imputation and complete case analyses, will be performed to evaluate the robustness of results. The secondary outcomes will be analyzed using appropriate mixed or generalized linear models and interpreted as exploratory. Descriptive statistics will summarize categorical variables as frequencies and percentages, and continuous variables as mean (SD), median (IQR), and range.

Predefined subgroup analyses will explore whether treatment effects differ by baseline Hoehn and Yahr stage (≤3.0 vs >3.0), disease duration (<5 vs ≥5 y), age (<65 vs ≥65 y), baseline LEDD (≤ median vs >median), and presence of dyskinesia. Interaction terms will be tested using mixed effects models, and the results will be interpreted as exploratory. Sensitivity analyses will include assessing participants with stable dopaminergic doses (<10% LEDD change) and testing the interaction between treatment allocation and LEDD change to explore medication-dependent effects. Safety analyses will be descriptive, summarizing AEs and serious AEs by treatment group.

### Monitoring and Oversight

DSMB constituted by the CCRAS, comprising experts in neurology, clinical trials, ethics, and Ayurveda, to oversee participant safety and trial integrity. The NIMHANS Ethics Committee will provide continuous ethical oversight. CCRAS will conduct independent monitoring for protocol, GCP, and regulatory compliance, ensuring objectivity. As an exploratory trial, no interim analysis is planned. However, the DSMB will periodically review safety data, including SAEs and potential risks, to ensure participant well-being. The DSMB, in consultation with the Institutional Ethics Committee and CCRAS, will make independent recommendations regarding trial continuation, modification, or termination. This process ensures ongoing participant safety and upholds the trial’s scientific and ethical integrity through consistent monitoring and safety oversight.

### Harms: AE Collection, Assessment, Reporting, and Management

Participants will be assessed for AEs at each scheduled follow-up visit (days 60, 120, and 180) and during routine hospital visits. Each reported AE will be documented and categorized based on severity, relatedness to the intervention, and expectedness, following standardized guidelines. Laboratory safety assessments, including liver function tests, RFTs, and complete blood count, will be performed at baseline and at the final study visit (day 180) to monitor any potential systemic effects. All recorded AEs will be reviewed by the DSMB, which will assess their clinical significance and provide recommendations for participant safety. SAEs will be promptly reported to the NIMHANS Human Ethics Committee and the sponsor, CCRAS, in accordance with regulatory guidelines.

For participants who discontinue or deviate from the intervention protocol, no further outcome assessments will be conducted, as the primary outcome (MDS-UPDRS Part III) requires evaluation in both ON and OFF medication states, and other assessments (TMS, pulmonary function tests, Montreal Cognitive Assessment, 6-m walk test, and Balance Error Scoring System) are performed in the OFF state. The reasons for nonadherence (eg, adverse effects, lack of perceived benefit, or personal constraints) and nonretention (eg, withdrawal or loss to follow-up) will be systematically documented either during the next scheduled visit or through telephonic communication. These details will be recorded in the case report forms to enable transparent reporting and analysis of participant flow and data completeness.

### Protocol Amendments

The PI will be responsible for communicating all significant protocol amendments. Substantial amendments will require approval from the accredited medical ethics committee before implementation, whereas nonsubstantial amendments will be documented and filed without separate notification. Any modifications impacting participant safety or study procedures will be promptly conveyed to enrolled participants, and, if necessary, a new informed consent process will be initiated.

### Protocol Version

The study was sanctioned on April 9, 2021 (3-29/2021- CCRAS/Admn/Coll).

## Results

As of August 2025, a total of 259 patients with PD have been screened for eligibility, of whom 58 participants have been successfully enrolled in the trial; among these, 33 participants have completed the intervention and follow-up assessments, 14 have discontinued participation, and 11 are currently continuing in the study. Recruitment and follow-up are ongoing, and the trial is scheduled for completion in September 2026.

### Plan to Disseminate Trial Results

The study results will be submitted for publication in peer-reviewed, open-access journals and presented at relevant national and international conferences. A summary of trial outcomes will also be posted on the Clinical Trial Registry of India. Dissemination materials will be jointly reviewed and approved by the PIs, the CCRAS, and the NIMHANS before submission or public release. The anticipated time frame for dissemination is within 12 months following study completion and final data verification.

## Discussion

### Anticipated Findings

The biomechanisms of conventional medicines are well established. The biosynthesis and metabolism of dopaminergic drugs, dopamine agonists, monoamine oxidase B inhibitors, catechol-O-methyl transferase inhibitors, anticholinergics, and amantadine used in the management of PD are established and well understood [8]. Levodopa-based preparations are designed to replace the dopamine in the depleted striatum. Levodopa is able to cross the blood-brain barrier and converts into the neurotransmitter dopamine by DOPA decarboxylase [28]. Dopamine agonists stimulate the activity of the dopamine system by binding to dopaminergic receptors. Monoamine oxidase B inhibitors work by inhibiting the enzymes involved in dopamine metabolism, which preserves the levels of endogenous dopamine [29]. Catechol-O-methyl transferase inhibitors offer a therapeutic means of preserving endogenous dopamine levels by reducing its breakdown [30]. Anticholinergics act through nondopaminergic mechanisms. These reduce the activity of the neurotransmitter acetylcholine by acting as antagonists at cholinergic receptors and offer some benefits in improving rigidity and tremor [31]. Amantadine acts as a weak glutamate antagonist at the N-methyl-D-aspartate receptor and is useful in the treatment of rigidity and rest tremor, and it can limit the severity of levodopa-induced dyskinesias [32].

Despite Ayurveda’s long-standing clinical application in managing neurodegenerative disorders, there is a lack of robust scientific evidence elucidating its mechanisms of action in comparison to conventional medicine. This study aims to bridge this gap by systematically evaluating the efficacy and safety of add-on Ayurveda therapy in PD and exploring its potential biomechanisms. By incorporating clinical, neurophysiological, immunological, and autonomic function assessments, this trial is expected to provide scientific validation for Ayurveda-based interventions in PD management.

The findings from this study could serve as critical evidence supporting the integration of Ayurveda in contemporary neurological care, potentially influencing clinical guidelines and policy decisions related to neurodegenerative disorders. Moreover, this research will provide preliminary data to guide future large-scale, multicentric trials and mechanistic studies exploring Ayurveda’s role in neuroprotection and disease modification. Establishing the scientific basis for Ayurveda in PD management may also contribute to its global acceptance, fostering interdisciplinary research and collaboration in integrative neurology.

### Ancillary and Posttrial Care

Participants completing the trial will continue to receive routine medical care through the OPD of NIMHANS. Clinical trial insurance has been secured to provide coverage for any unforeseen AEs, ensuring comprehensive risk mitigation and participant safety.

## Supplementary material

10.2196/83336 Multimedia Appendix 1 Patient information sheet and consent forms.

10.2196/83336 Checklist 1 SPIRIT checklist.

## Abbreviations

AE: adverse event
CAM: complementary and alternative medicine
CCRAS: Central Council for Research in Ayurvedic Sciences
DSMB: Data and Safety Monitoring Board
GCP: Good Clinical Practice
HRV: heart rate variability
ICF: intracortical facilitation
LEDD: levodopa equivalent daily dose
LICI: long-interval cortical inhibition
MDS-UPDRS: Movement Disorder Society–Unified Parkinson’s Disease Rating Scale
MT: motor threshold
NIMHANS: National Institute of Mental Health and Neurosciences
OPD: outpatient department
PD: Parkinson disease
PI: principal investigator
RCT: randomized controlled trial
SICI: short-interval cortical inhibition
SP: silent period
SRF: senior research fellow
TAU: treatment as usual
TMS: transcranial magnetic stimulation

## References

[1] Jankovic J (2008). Parkinson’s disease: clinical features and diagnosis. J Neurol Neurosurg Psychiatry.

[2] Pearce JM (1989). Aspects of the history of Parkinson’s disease. J Neurol Neurosurg Psychiatry.

[3] Singhal B, Lalkaka J, Sankhla C (2003). Epidemiology and treatment of Parkinson’s disease in India. Parkinsonism Relat Disord.

[4] Razdan S, Kaul RL, Motta A, Kaul S, Bhatt RK (1994). Prevalence and pattern of major neurological disorders in rural Kashmir (India) in 1986. Neuroepidemiology.

[5] Bharucha NE, Bharucha EP, Bharucha AE, Bhise AV, Schoenberg BS (1988). Prevalence of Parkinson’s disease in the Parsi community of Bombay, India. Arch Neurol.

[6] Cerri S, Mus L, Blandini F (2019). Parkinson’s disease in women and men: what’s the difference?. J Parkinsons Dis.

[7] Padovani A, Costanzi C, Gilberti N, Borroni B (2006). Parkinson’s disease and dementia. Neurol Sci.

[8] Church FC (2021). Treatment options for motor and non-motor symptoms of Parkinson’s disease. Biomolecules.

[9] Müller T, Russ H (2006). Levodopa, motor fluctuations and dyskinesia in Parkinson’s disease. Expert Opin Pharmacother.

[10] Nutt JG (2001). Motor fluctuations and dyskinesia in Parkinson’s disease. Parkinsonism Relat Disord.

[11] Pandit AK, Vibha D, Srivastava AK, Shukla G, Goyal V, Behari M (2016). Complementary and alternative medicine in Indian Parkinson’s disease patients. J Tradit Complement Med.

[12] Chikkanna U, Venkatram S, Holla B (2025). Exploring Ayurveda’s potential in Parkinson’s disease: a comprehensive narrative. Cureus.

[13] Agnivesa (2017). Charaka Samhita.

[14] (2024). Subject: draft guideilnes on good clinical practices - regarding. ICMR Bioethics Unit.

[15] Rizzo G, Copetti M, Arcuti S, Martino D, Fontana A, Logroscino G (2016). Accuracy of clinical diagnosis of Parkinson disease: a systematic review and meta-analysis. Neurology (ECronicon).

[16] Takahashi M, Shimokawa T, Koh J (2022). Efficacy and safety of istradefylline in patients with Parkinson’s disease presenting with postural abnormalities: results from a multicenter, prospective, and open-label exploratory study in Japan. J Neurol Sci.

[17] World Medical Association (2013). World Medical Association declaration of Helsinki: ethical principles for medical research involving human subjects. JAMA.

[18] Goetz CG, Stebbins GT, Tilley BC (2012). Calibration of unified Parkinson’s disease rating scale scores to movement disorder society-unified Parkinson’s disease rating scale scores. Mov Disord.

[19] Goetz CG, Stebbins GT, Chung KA (2013). Which dyskinesia scale best detects treatment response?. Mov Disord.

[20] Martínez Martín P, Cubo Delgado E, Aguilar Barberà M (2006). Estudio piloto sobre una medida específica para los trastornos del sueño de la enfermedad de Parkinson: SCOPA-Sueño [A pilot study on a specific measure for sleep disorders in Parkinson’s disease: SCOPA-Sleep]. Rev Neurol.

[21] Nasreddine ZS, Phillips NA, Bédirian V (2005). The Montreal Cognitive Assessment, MoCA: a brief screening tool for mild cognitive impairment. J Am Geriatr Soc.

[22] Kobayashi E, Himuro N, Takahashi M (2017). Clinical utility of the 6-min walk test for patients with moderate Parkinson’s disease. Int J Rehabil Res.

[23] Duncan RP, Combs-Miller SA, McNeely ME (2017). Are the average gait speeds during the 10 meter and 6 minute walk tests redundant in Parkinson disease?. Gait Posture.

[24] Bell DR, Guskiewicz KM, Clark MA, Padua DA (2011). Systematic review of the balance error scoring system. Sports Health.

[25] Kim HM, Nazor C, Zabetian CP (2019). Prediction of cognitive progression in Parkinson’s disease using three cognitive screening measures. Clin Park Relat Disord.

[26] Udupa K (2021). Transcranial magnetic stimulation in exploring neurophysiology of cortical circuits and potential clinical implications. IJPP.

[27] Central council for research in ayurvedic sciences. https://www.ccras.nic.in.

[28] Beckers M, Bloem BR, Verbeek MM (2022). Mechanisms of peripheral levodopa resistance in Parkinson’s disease. NPJ Parkinsons Dis.

[29] Riederer P, Müller T (2018). Monoamine oxidase-B inhibitors in the treatment of Parkinson’s disease: clinical-pharmacological aspects. J Neural Transm (Vienna).

[30] Rivest J, Barclay CL, Suchowersky O (1999). COMT inhibitors in Parkinson’s disease. Can J Neurol Sci.

[31] Gonzalez-Latapi P, Bhowmick SS, Saranza G, Fox SH (2020). Non-dopaminergic treatments for motor control in Parkinson’s disease: an update. CNS Drugs.

[32] Carrillo-Mora P, Silva-Adaya D, Villaseñor-Aguayo K (2013). Glutamate in Parkinson’s disease: role of antiglutamatergic drugs. Basal Ganglia.

