# Supplementary material for: Ayurvedic Therapeutic Regimen as an Add-On to Optimized Conventional Management of Parkinson Disease: Protocol for an Exploratory Randomized Controlled Trial Evaluating Clinical, Cortical Excitability, Neuroimmune, and Autonomic Function Parameters
Source: JMIR Res Protoc. 2026 Jan 9;15:e83336. doi: 10.2196/83336 (PMC12788784; doi:10.2196/83336)
Supplement: Multimedia Appendix 1 [file resprot-v15-e83336-s001.pdf]

PATIENT INFORMATION SHEET

Title of the Study

"Ayurveda therapeutic regimen as on Add-on to optimized conventional management of Parkinson's disease: An RCT for assessment of Clinical, Cortical excitability Neuroimmune and Autonomic function parameters"

1. Invitation

You are being invited to take part in a research study. Before you decide it is important for you to understand why the research is being done and what it will involve; please take time to read the following information carefully and discuss it with friends and relatives if you wish. Ask us if there is anything that is not clear or if you would like more information. Take time to decide whether or not you wish to take part. You should not sign this form until you understand all of the information presented in the following pages and until all of your questions about the research have been answered to your satisfaction.

2. What is the purpose of the study?

This is a research study being conducted at National Institute of Mental Health and Neurosciences, Bengaluru and is funded by CCRAS, Ministry of AYUSH Govt. of India, involving administration of Mashabaladi kvātha, Kalyanaka Gritha, Chitrakadi Vati and panchakarma therapies Abhyanga (therapeutic massage) and Basti (Medicated enema) along with optimized standard treatment in diagnosed cases of Parkinson's disease.

Recruitment of the subjects will be started only after submission to CTRI.

3. Why have I been chosen?

Being a patient of Parkinson's disease, you are considered as an ideal candidate for the study.

4. Do I have to take part?

It is up to you to decide whether or not to take part. If you do decide to take part you will be given this information sheet to keep and be asked to sign a consent form later. If you agree to take part you are still free to withdraw at any time and without giving any reason. This will not affect the standard of care you receive.

5. What will happen to me if I take part?

If you agree to take part in this study, you will be one of the 80 subjects we plan to recruit in this study. You will be assigned to either of the two study treatment arms. One group of patients will receive Standard care as decided by the neurologist, for 6 months, while other group of patients will receive Mashabaladi kvātha, Kalyanaka Gritha (every 2 months for 47 days X 3 cycles), Chitrakadi Vati (every 2 months for 5 days X 3 cycles), therapeutic massage and medicated enema (every 2 months for 8 days X 3 cycles) along with optimized standard care as decided by neurologist. The total duration of the study will be 180 days. You will be assigned

to treatment as usual arm or add-on Ayurveda therapy arm by randomization. Randomization means that you will be assigned to treatment as usual or add-on Ayurveda therapy by chance, like flipping a coin etc. There will be 50-50 chance for you to receive either treatment as usual or add-on Ayurveda therapy. Randomization will be done by random number generator at www.randomization.com. Neither you or your doctor can choose which treatment you will receive. Subjects in treatment as usual arm will be offered add-on Ayurveda therapy after the completion of the trial. You may have to agree to provide blood sample about 10ml. These samples will be taken before the start of the treatment and at the end of the treatment. The sample collected will be tested to see the changes in Th1/Th2/Th17/T regulatory cell population, plasma levels of pro-inflammatory cytokines and telomere length. Further, Possible drug interactions (Anti-Parkinson and Ayurveda drugs) and the safety aspects of the trial drugs will be checked using routine blood investigations like complete hemogram, lipid profile, Liver function test, and Renal function test. The possible risk of blood sampling includes occasional pain, bruising or fainting. All due aseptic precautions will be taken during the procedures, these procedures will be conducted by qualified and competent experts. Heart rate variability (HRV) and Pulmonary function tests (PFT) will be done for evaluating Autonomic functions. Trans cranial magnetic stimulation (TMS) will be done to know the cortical excitability. HRV consists of recording surface ECG for 5 to 10 mins and PFT consists of blowing of the air through mouth piece of spirometer. HRV will not have any risk if proper procedure is followed, whereas slight dizziness, shortness of breath and cough may be experience during PFT, by following the proper procedures these can be prevented/minimized. Mild head ache, mild tinnitus in the ear due to the sound produced by magnetic coil placed on head and in the process of trying to locate the motor hot spot can be experienced during TMS. Mild headache will be relieved by taking rest or over the counter pain-killers and tinnitus can be avoided reduced or by providing ear plugs before recording. The investigator can adjust the level of stimulation to reduce symptoms and are generally very mild and improve shortly after the session. Apart from this the doctor will ask about your health status and check clinical signs as a part of general physical examination. All the tests and Ayurveda therapies will be conducted as In-patient, free of cost and specific advice about taking medicines and diet will be given.

6. What do I have to do?

You have to adhere to the instructions given to you by your investigating physician regarding taking the medicines as advised and follow up on the prescribed day. During the course of the trial, you can continue to take your regular medication for any other health condition after informing your investigating physician. You are requested to follow the instructions of your investigator while continuing with the trial drugs.

7. What is the drug or procedure that is being tested?

The Ayurveda medicines Mashabaladi Kvātha, Kalyanaka Gritha, Chitrakadi Vati and therapeutic massage and medicated enema therapies are the drugs and procedures being tested. These Ayurveda medicines and therapeutic procedures are routinely prescribed by an Ayurveda physician for management of Parkinson’s disease either as standalone treatment/ as adjunct to conventional therapy.

8. What are the alternatives for diagnosis or treatment?

Diagnosis is made based on Diagnostic criteria of Parkinson's disease according to UK Parkinson's disease society brain bank clinical diagnostic criteria (UKPDSBB) and Ayurvedic Diagnosis of kampavāta. Other treatment/medicinal options that are available to treat Parkinson's disease include levodopa/carbidopa, dopamine agonists, monoamine oxidase-B (MAO-B) inhibitors, injectable dopamine agonist (apomorphine), catechol-O-methyltransferase (COMT) inhibitors, N-methyl-Daspartate (NMDA) receptor inhibitors, and anti-cholinergics. Invasive procedures like Deep brain stimulation are also employed as palliative treatment.

9. What are the expected side effects / risks of the treatment?

The Ayurvedic formulations and therapeutic procedures being prescribed to you have been used as remedy for Parkinson's disease by the Ayurveda physicians as standalone or as adjunct along with other conventional therapy medicines. They have been found to be safe and effective. Possible drug interactions (Anti-Parkinson and Ayurveda drugs) and the safety aspects of the trial drugs will be checked using routine blood investigations like complete hemogram, lipid profile, Liver function test, and Renal function test. Appropriate clinical support will be provided in case of any adverse event. Any adverse event arising out of the test drug only will be covered under insurance.

10. What are the possible benefits of taking part?

Your participation will help us in generating sufficient data to validate the efficacy and safety of Mashabaladi kvātha, Kalyanaka Gritha, Chitrakadi Vati and panchakarma therapies Abhyanga (therapeutic massage) and Basti (Medicated enema) in managing/treating Parkinson's disease. The results of the research may provide benefits to the society in terms of advancement of medical treatments and / or therapeutic benefit to future patients.

11. What if new information becomes available?

If during the course of the clinical trial some new information becomes available about the Ayurvedic treatment being studied, you will be informed about that by your investigating physician after which you are free to decide whether you want to continue in the study or not. If you decide to withdraw, this will not at all affect your routine care in the hospital. If you decide to continue in the study, you will be asked to sign a fresh consent form. On the other hand, upon receiving new information your investigating physician might consider it to be in your best interests to withdraw you from the study. Your investigating physician will explain the reasons for dropping you from the study and arrange for your routine care to continue.

12. What happens when the research study stops?

You will be given appropriate advice for future line of treatment.

13. What if something goes wrong?

Your safety is the prime concern of the research study. In case of any adverse event Appropriate clinical support will be provided. Further, the adverse event will be evaluated by a committee of experts belonging to Ayurveda system and Modern Medical science and if established that the adverse event has happened due to investigational medicines, you will be compensated through Insurance.
Contact address of the Investigators (Doctors)

1. Dr Umesh C, (Ayurveda Expert), Scientist - C (Ayurveda), Department of Integrative Medicine, NIMHANS Bengaluru; +91 7975856612 (Mob); Email drumeshayu@gmail.co
2. Dr Nitesh Kamble, Associate Professor, Department of Neurology, NIMHANS, Bengaluru; +91 9886800609; email – nitishlk@gmail.com

14. Will my taking part in this study be kept confidential?

Yes, all your information will be kept confidential but any of your medical records may be inspected by the Sponsors for the purpose of analysing the results. They may also be looked at by members of Institutional Ethics Committee and by Regulatory authorities / court to check that the study is being carried out correctly. Your name, however, will not be made public and any sensitive matter regarding your state of health will be kept confidential.

15. What will happen to the results of the research study?

The results of the clinical trial will be published in leading medical journals so that other doctors and researchers can benefit from the results. You can ask your investigating physician for a copy of the publication. If published, your identity and personal details will be kept strictly confidential. No named information about you will be published in any of the trial reports.

16. Who is organizing and funding the research?

The Research Study has been funded by CCRAS, Ministry of AYUSH, Govt. of India, New Delhi.

17. Contact for further information

If desirous of any relevant information at any stage of the clinical trial, you may feel free to ask your investigating physician for that information. You would be given a copy of the information sheet and a signed consent form

Consent Form

“Ayurveda therapeutic regimen as on Add-on to optimized conventional management of Parkinson’s disease: An RCT for assessment of Clinical, Cortical excitability Neuroimmune and Autonomic function parameters”

Centre: National Institute of Mental Health and Neurosciences, Bengaluru.

I certify that I have disclosed all details about the study in the terms easily understood by the subject. Your consent to participate in the above study is sought. You have the right to refuse consent or withdraw the same during any part of the study without giving any reason. In such an event, you will still receive best possible alternative treatment, without any prejudice. If you have any doubts about the study, please feel free to clarify the same. Even during the study, you are free to contact any of the investigators for clarification if you so desire (investigator Name, Department and contact Telephone No. need to be furnished). All the information/data collected from you (participant) will be kept in strict confidence.

CONSENT BY PARTICIPANT

1. I confirm that I have read / the study and has been explained to me adequately and I have understood the information for the above study and had the opportunity to ask questions.
2. I hope to complete the study, but I understand that my participation is voluntary and that I am free to withdraw at any time, without giving a reason, and without my medical care or legal rights being affected.
3. I understand that my doctor will provide information about my progress, in confidence, to the related officers of the participating Institutions and CCRAS, Ministry of AYUSH, Govt. of India. I understand that the information held by the Investigators and researchers and records might be used to follow up my health status.
4. I understand that the information will be used for medical research only and that I will not be identified in any way in the analysis and reporting of the results. I understand that sections of any of my medical notes may be looked at by the Sponsors or responsible individuals of the Institutional Ethics Committee (IEC), Regulatory authorities or Court, if necessary. I give permission for these individuals to have access to my records.
5. I understand what is involved in this trial and agree to take part in the clinical trial for a period of 180 days (including follow up period.).
6. I am aware that by subjecting to this research, I will have to give more time for assessments by the investigating team and that these assessments do not interfere with the benefits.

Name of participant	Signature/ Thumb impression	Date
Name of investigator	Signature/ Thumb impression	Date
Name of the witness	Signature/ Thumb impression	Date
